# Mental health related stigma in Romania: systematic review and narrative synthesis

**DOI:** 10.1186/s12888-023-05147-3

**Published:** 2023-09-08

**Authors:** Elena A. Manescu, Claire Henderson, Ciprian R. Paroiu, Adriana Mihai

**Affiliations:** 1 Department of Psychiatry, George Emil Palade University of Medicine, Pharmacy, Science, and Technology of Targu Mures, Tirgu-Mures, Romania; 2 Department of Health Service and Population Research, King’s College London Institute of Psychiatry, Psychology and Neuroscience, London, UK; 3 South London and Maudsley NHS Foundation Trust, London, UK

**Keywords:** Stigma, Mental illness, Review, Romania

## Abstract

**Background:**

Stigma related to mental illness is increasingly and more effectively addressed. Although more research is being conducted, there is relatively little from low and middle-income countries, with former Soviet bloc countries identified as a particular contributor to this evidence gap. Romania struggles with mental health stigma at many levels. The aim of this review was to bring together all relevant data regarding stigma and discrimination related to mental illness as well as actions to address these problems in Romania in order to prioritise further stigma research and identify priority targets for stigma reduction.

**Methods:**

A systematic literature search was conducted in three online databases and grey literature. After the analysis of full manuscripts, four were excluded based on lack of relevance or incomplete data reporting. Quality assessment was performed for included studies using the Mixed Methods Appraisal Tool (MMAT) and the narrative was synthesized based on the research questions.

**Results:**

The review included fifteen studies, the majority having a cross-sectional design. Stigma related to mental illness in Romania, has an impact on help-seeking attitudes and behaviours, workplace environment and social relationships of people with mental health problems. Negative stereotypes are maintained and validated by mass media reports. Significant stigma in healthcare and mental care settings has also been observed. Self-stigma was less frequently reported than public stigma. Despite a few stigma reduction actions, no rigorous evaluation of content, delivery and effectiveness has been conducted and no empirical evidence has been published.

**Conclusions:**

Based on this review, three lines of action are recommended in Romania. Increase research concerning stigma in healthcare and mental care settings and use behavioural outcomes. Develop and deliver evidence-based tailored interventions to reduce stigma in identified priority subgroups of the population and ensure rigorous evaluation and scientific dissemination. Elaborate guidelines for working with community stigma and advocate for structural changes.

**Supplementary Information:**

The online version contains supplementary material available at 10.1186/s12888-023-05147-3.

## Background

Winkler et al. [1] drew attention to what they described as a blind spot on the global mental health map in 2017, referring to countries in central and Eastern Europe. One of their findings was that stigma appears to be higher than in other European countries. In general, greater recognition of stigma related to mental illness has been observed in recent years along with more effort being made to reduce it [2]. Researchers have reported several negative effects of stigma such as reluctance to seek help, engage in and maintain treatment [3], and an overall increase in co-morbidity and mortality [4, 5]; fewer opportunities for school, work or social interactions or trouble obtaining accommodation [6, 7]. In response to these concerns, the World Health Organization and many other international alliances have highlighted the importance of reducing both public and structural stigma through anti-stigma interventions and advocacy for policy change [2, 8]. National anti-stigma campaigns or smaller-scale interventions focused on several target groups have been conducted mainly in developed high-income countries while there is less evidence coming from low and middle income countries (LMICs) [2, 9, 10]. These findings suggest a clear and strong need for a coordinated effort to conduct more high-quality research, particularly in LMICs in order to provide further evidence to support policy decisions [2] In the context of local evidence on stigma, it is important for central and eastern European countries, and in particular for Romania, to consider the recommendations of the experts’ commission on ending stigma and discrimination in mental health [2].

The transition from the communist regime that embraced central and Eastern Europe at the beginning of the nineties has found Romania not optimally adjusted to the new democracy. This fact is also reflected in the evolution and development of the mental health system. Although constrained to adapt to the European standards since 2007, the legislation regarding mental health in Romania was in fact modified and updated more in theory and less in practice. Examples are numerous but some deserve special attention being related to the problem discussed here. Structural stigma in the Romanian mental healthcare system comes from many levels. For instance, the national mental health legislation stipulates a general deinstitutionalization of the psychiatric service and a greater focus on the community level [11]. The number of beds in psychiatric hospitals has been cut progressively in the last years but without an accompanying development of community services [1, 10]. In fact, Romania has been criticized for the alarming underdevelopment in this area compared to other eastern European countries [1, 10]. Community services are currently available in a few regions of the country and even fewer centres are efficiently communicating with other mental health organizations, resulting in discrimination against people with mental illness in relation to access to care.

Another stigma-generating context is represented by the low level of implementation of liaison psychiatry into the National Healthcare System. Despite being one of the first countries of the former communist block to create and implement such a department in a general hospital in 1995 [12], the further development of this subspecialty did not take place as expected due to poor allocation of funds and a general lack of coordination between parties involved in the decision-making. The Romanian National Mental Health Reform Act [11] provides for the establishment of liaison psychiatry departments since 2002. Still, only small steps have been taken to include psychiatric beds in general hospitals especially in small towns, the initiative being progressively abandoned. Under these circumstances, the quality of treatment for people with psychiatric related problems can be significantly affected. On the other hand, psychiatric patients are often discriminated against by other health specialists who neglect their medical responsibilities towards these patients by avoiding professional contact with them.All these realities of the Romanian health system suggest a significant level of both structural and public stigma insufficiently addressed by policy and executive changes.

There have been a few uncoordinated anti-stigma actions mainly supported by international sources, with reduced activity in recent years, similar to other initiatives recommended by the national mental health reform [11].

Before these problems can be tackled, we need to have a clear and detailed image of the stigma-related background in Romania. To the best of our knowledge, there has been published no review work that comprehensively summarizes the current evidence regarding stigma related to mental illness in Romania. To address this gap, we conducted a systematic review of the stigma research related to Romania and provide a conceptual overview of the evidence for the development of a future intervention targeting mental health professionals. The paper highlights the most important results by addressing the following questions: “How has mental illness stigma in Romania been studied?”, “What are the socio-demographic factors associated with mental health stigma?”, “What differences does comparative research on stigma including Romania suggest??” and “What interventions have been used to reduce mental health-related stigma in Romania?”. Based on the evidence, we draw recommendations for research, policy and practice.

## Methods

The review was conducted according to the Preferred Reporting Items for Systematic Reviews and Meta-Analyses (PRISMA) checklist recommended for systematic reviews [13], as outlined in Additional file 1. The No ethical approval was sought for this study as it involved analysis of previously published report.

A scoping search of the literature was performed to get on overview of the existing research on mental health related stigma in Romania. Three databases were searched, Web of Science, Google Scholar and MEDLINE, from their start date to the search date (March 2021). The results were a small number of studies mostly covering manifestations of stigma in specific supgroups of the general population and some targeting anticipated or perceived/experienced stigma and discrimination. Beyond the overall scarcity of data, already reported in a recent scoping review [1], there was a gap of evidence regarding stigma reduction interventions and stigma in healthcare and mental healthcare settings. No review of the literature was found on the searched topic.

### Information sources

Three different scientific databases: Scopus, Web of Science and MEDLINE, were searched from their start date to the search date (20^th^ October 2021 and updated in 12^th^ September 2022) for articles in English and Romanian language. An additional Google Scholar search was made to identify relevant grey literature. Grey literature was also searched from OpenGrey database and via the Entireweb metasearch engine for other potential sources, including technical or research reports from government agencies, reports from scientific research groups, doctoral dissertations and conference abstracts. We also conducted searches of key authors’ article lists and used Google Scholar to find studies listed by the author but not identified in the database search. References in relevant articles were screened for publications that might be acceptable for inclusion. We consulted mental health stigma experts in Romania for the potential inclusion of other articles. The search terms were kept wide and no restrictions were applied for publication type.

### Search strategy (Additional file 2)

A search strategy was created based on the preliminary exploration and after consulting a specialist. (for full database search strategies, please check the Additional file 2).

Two sets of terms were used in the search: stigma terms (“stigma”, “discriminat$”, “stereotyp$”, “attitud$”, “social behavio$”) and mental disorder terms (“mental illness$”, “mental disorder$”, “schizophrenia”, “depression”). In addition, Medical Subject Headings (MeSH) terms, keywords, and their synonyms were used to develop the search strategy for PubMed and adapted for the other databases (Additional file 2). Following the literature search, the references were exported to an EndNote database and the duplicates removed.

The following search strategy was used:

((‘Stigma terms’ tw AND ‘mental disorder terms’ tw) OR (stigma MESH terms AND mental disorder MESH terms) AND Romania).

### Inclusion and exclusion criteria

Search strategy and selection of articles was based on the SPIDER (Sample, Phenomenon of interest, Design, Evaluation, Research type) framework, chosen because of its suitable application to qualitative and mixed methods research [14]. We included articles targeting the Romanian general population or subgroups, mental health service users and mental health professionals. Studies that included a broader study sample (e.g. international multicentre studies) were included if the results for Romanian sample were reported separately or can be distinguished in the results. Included studies explored any form of anticipated, perceived or experienced stigma in relation to mental illness (e.g. knowledge, attitudes, behaviours) identified as a research question or aim, key theme or major finding in the results. Any study design was allowed, including descriptive reports and qualitative research based on focus groups and interviews. Outcomes relate to stigma in terms of knowledge, beliefs, attitudes and behaviours by either a quantitative or a qualitative method and can include other associated factor as secondary outcomes. As for research type, the review covered peer-reviewed studies published in English or Romanian language prior to September 2022 as follows: (i) quantitative studies, (ii) qualitative studies that used appropriate methods of data collection and (iii) mixed methods – studies combining both methods of data collection. Review articles, thesis and dissertations were also included, while articles not published in a peer reviewed journal or English and Romanian language, were excluded. Studies with outcome measures outside the common conceptualization of stigma were excluded as well as incomplete studies such as protocols.

### Study selection

**Fig. 1:**
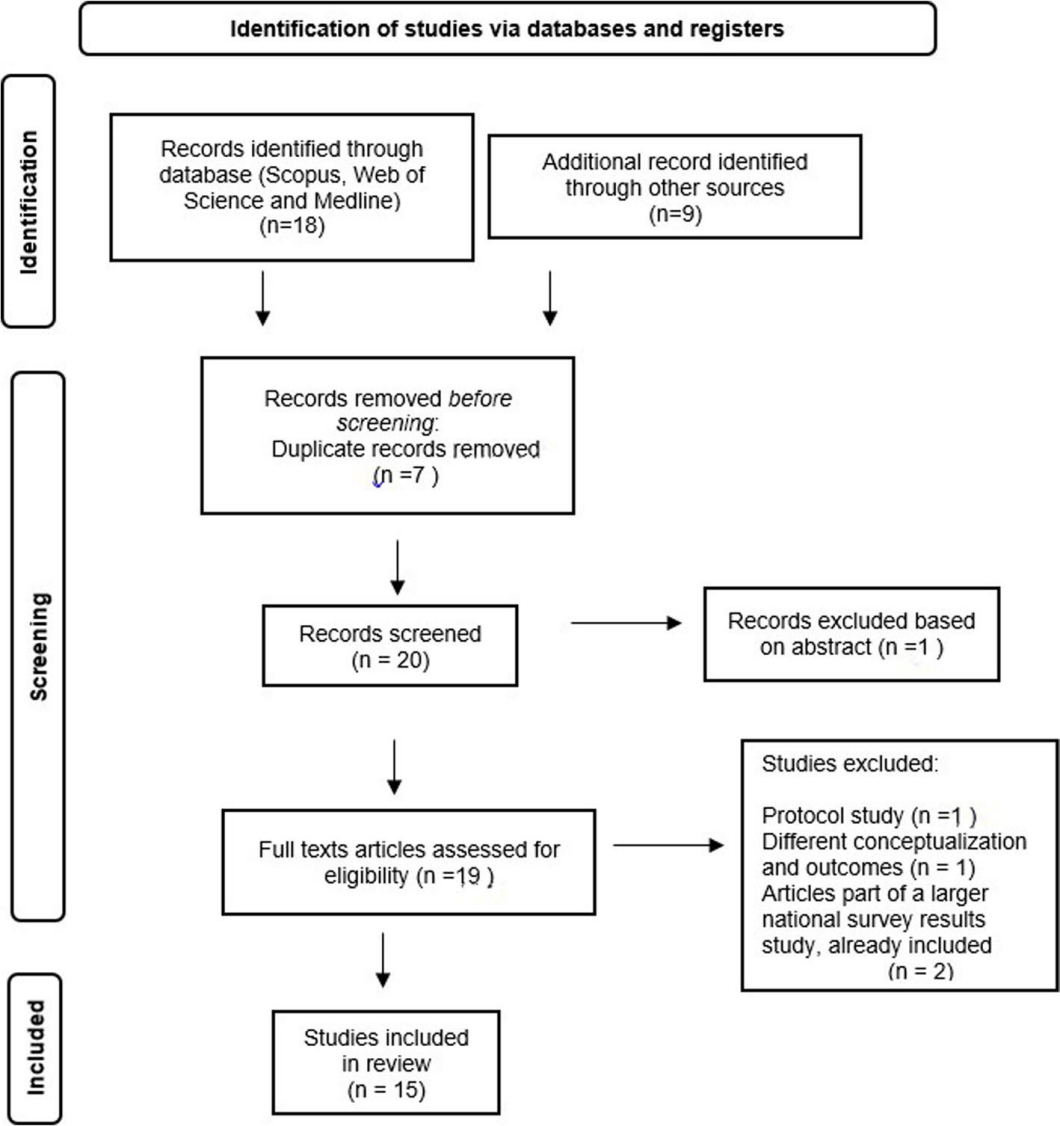
PRISMA flowchart study selection

The search was conducted by the first author (EM) across all databases and an initial screening was independently conducted by two authors (EM and CP) using the PRISMA Flowchart (Fig. 1). Each of the reviewers removed the duplicates in the initial screening process and potentially relevant full-text articles were obtained. The authors reviewed all studies based on the eligibility criteria, by reading all titles, followed by selected abstracts, full texts and references. In the occurrence of uncertainty whether an article met inclusion criteria, the first author consulted the senior authors. All disagreements were resolved by discussion and consensus with the senior reviewers CH and AM.

### Risk of bias and quality assessment

Assessment of bias and quality was done independently by two authors (EM and CP) and discussed until agreement was reached. We used the Mixed Methods Appraisal Tool (MMAT), version 2018 [15]. MMAT assesses the methodological quality of quantitative, qualitative and mixed methods studies, making it a suitable quality assessment tool for systematic mixed studies reviews as ours. The checklist covers seven quality criteria for each methodological design. The authors advise on reporting a descriptive summary of quality ratings rather than a scoring and elimination based on low quality rating is discouraged. As our rationale was to cover all of the literature relevant to this review and because of the small number of studies found, those relevant studies which did not meet all the seven criteria were not excluded.

### Data extraction and narrative synthesis

Results of the full text selection were shared with all the authors to validate their eligibility, before data extraction. The framework for data extraction included the following study characteristics: general information (authors and year of publication, study title and design), study population (sample size, target population), outcome measures corresponding to knowledge, attitudes, inteded behaviour components, anticipated, perceived and experienced stigma and discrimination, structural stigma (narrative descriptions or any other form), anti-stigma interventions (type, primary and secondary outcomes,duration of follow-up and evidence for effective interventions). Qualitative narratives and recommendations were collected from both qualitative and quantitative studies. After data extraction, the narrative synthesis was conducted based on the research questions and adapted from the guideline proposed by Popay et al. [16]. This guideline recommend four main elements: developing a theory on the concepts on which the research question is based; developing a preliminary synthesis of findings of included studies; exploring relationships in the data; and assessing the robustness of the synthesis. Extracted data was summarized using the tabular form of the Cochrane review’s [17] ‘Characteristics of Included Studies’ table (methods, participants, intervention, outcomes, notes). Research papers were imported into NVIVO 12 for narrative synthesis and coding was carried out of the included papers. Initial codes were generated by inductive reasoning from the material and these were then clustered to form themes.

## Results

### Characteristics of the studies

A total of nineteen papers were identified in the search and after initial screening, four were excluded: one study was a proposed protocol for an anti-stigma intervention [18]; one studied public beliefs of social versus medical etiology of mental illness [19] and was excluded as although etiological beliefs can influence stigma it was not a study of stigma as such; and the other two [20, 21] papers were part of a larger study included in the current review.

Most of the included studies (86%) were cross-sectional, either part of an international analysis of stigma associated with mental illness in several countries or national initiatives. Data reported on stigma comes from the general population and from mental health service users. Seven studies evaluated stigma in specific subgroups of the general population such as teenagers [22], medical and other college students [23–26] and healthcare professionals [27, 28]. Two studies used a representative sample of the general population aged 18 and over [29, 30]. The national research study [29] was based on a probabilistic, stratified national sample that included 1070 persons from 67 Romanian cities and communes, including Bucharest. The sample consisted of 44% respondents from rural areas and 56% urban respondents (11% from small towns, 12% from medium-sized towns and 32% from cities). The survey included in addition a group of 98 mental health experts. Qualitative data comes from interviews and focus groups carried out with mental health professionals, service users and the general population.

Four other studies reported the experience of mental health service users in terms of anticipated or experienced discrimination and self-stigma [31–34] and one looked at factors associated with delays in seeking professional help [35]. One study described anti-stigma interventions [36]. Most of the included studies were published more than ten years ago.

### Quality assessment

All fifteen studies were analysed based on the seven methodological quality criteria. The majority of the studies (86%) were quantitative descriptive (e.g. cross-sectional study, survey) in nature [22–28, 30–35]. One study used a mixed methods design and one study was a qualitative research. The studies met the seven quality criteria in general. The main shortcoming was that of non-reporting the response rate; 60% of the included studies did not report the response rate of the participants. For more information, see Additional file 3.

### How has mental illness stigma in Romania been studied?

Included studies evaluated stigma in its public and internalized aspects. Public stigma was reported in terms of attitudes towards people with mental illness and towards help-seeking. As stigma can be considered one aspect of mental health literacy (MHL) [37], results related to MHL will be presented in this section The patients’ perspective was reported as perceived, anticipated, and experienced stigma and discrimination and self-stigma. Structural stigma was reported as mental health professionals’ opinion.

#### Mental health literacy

Three studies evaluated mental health literacy either in specific subgroups of the population such as students [22, 24] or in the general population as is the case with the national community survey [29]. All the studies used a case vignette model with comparable accompanying questions about recognition, etiology and management of mental illness, extracted from international referential research (see Table 1). Students in tenth grade [22] scored low in MHL; only a few correctly identified the case vignette depicting schizophrenia (17.7%), while more than half considered it to be a psychological problem (51.7%) rather than a medical and psychological problem (39.2%). College students were compared based on their nationality as either Romanian students or other nationality students [24]. The majority of students in both subgroups recognized the vignette depicting schizophrenia as being a mental illness but almost half (46.6%) of the Romanian students did not recognize depression as such and almost a quarter (23.3%) of all students did not consider alcohol dependence as being a mental illness. Similar results are reported in the community: almost half of the participants did not consider depression (49.8%) and alcohol dependence (49.6%) as being a mental illness while schizophrenia was recognized as such by most of participants (74.6%) [29]. Lack of knowledge regarding mental illness is greater however in those with low educational level and elderly people, especially in rural areas [29].

**Table 1:** Characteristics, main results and quality assessment scores of the studies reviewed

Reference	Design	Characteristics of participants (N)	Outcome measures	Main results	Quality assessment MMAT ratings
Stanculescu et al. (2008) [29]	Cross-sectional survey	National representative sample of the general population aged 18 years and over; (1070) Mental health experts; (98) Service users;	Vignettes from the MacArthur Mental Health Social Survey model covering four case vignettes (alcohol addiction, major depressive disorder, schizophrenia and a control- a mentally healthy individual that goes through hardships lately) [38]; interviews and focus groups with mental health professionals, service users and the general population; Stigmatization of people with mental illness was measured by replicating a study of Schomerus et al. [39], carried out in three locations (Slovakia, Germany and Russia). The study used the perceived devaluation and discrimination (PDD) scale developed by Link et al. (1987) [40]	Romanian population is deficient regarding specific mental health terms but has good overall recognition of mental health problems that need psychiatric intervention and differentiation of multiple causes of mental illness More than two-thirds of the population held negative stereotypes of dangerousness towards people with mental illness, especially towards schizophrenia and alcohol addiction while people with depression are more easily tolerated. People who have been hospitalized are in particular more stigmatized, the majority of the population having attitudes of devaluation and discrimination towards these people. Negative attitudes are maintained and enhanced by the mass media Stigma and discrimination are higher in Romania compared to regions in Germany, Slovakia and Russia and affect most severely the labour and marital market. Stigma is also present in family, friends and healthcare professionals who frequently delay or avoid contact with psychiatric patients	7/7 QA items
Euro-barometer survey (2006) [30]	Cross-sectional survey	National representative sample of the general population aged 15 years and over (1003)	Four items questionnaire regarding attitudes towards people with emotional and psychological problems	65% of the Romanian population believe people with mental health problems are unpredictable, 43% think they represent a danger to others, 20% believe they will never recover and 14% think they are to blame for their psychological problems. Stigmatizing attitudes were slightly more common in the Romanian population compared to the European average	7/7 QA items
Evans-Lacko et al. (2012) [31]	Cross-sectional	General population –Eurobarometer survey in 14 countries (1003) Mental health service users in 14 European countries (1835)	Internalized Stigma of Mental Illness Scale (ISMI) [41]; Perceived Devaluation and Discrimination Scale (PDD) [40]; Boston University Empowerment Scale [42] (BUES); a scale with 4 items for assessing attitudes; Questionnaire evaluating help-seeking, access to information, antidepressant use and comfort when talking to someone with a mental health problem	People with mental illness living in countries with less stigmatizing attitudes, higher rates of help-seeking and treatment use, and better-perceived access to information reported lower rates of self-stigma and perceived discrimination but not higher empowerment. Those living in countries where the public felt more comfortable talking to people with mental illness had lower levels of self-stigma and felt more empowered Being employed and having a higher educational level were associated with less self-stigma Less perceived discrimination was reported by individuals with depression compared to those with schizophrenia and women reported higher levels of perceived discrimination than men. Public attitudes endorsing recovery pessimism were associated with higher perceived discrimination by service users Being employed and having a higher educational level were associated with higher empowerment scores. Women, however reported lower empowerment scores	7/7 QA items
Brouwers et al. (2015) [32]	Cross-sectional	People diagnosed with major depression in the previous year (834)	The Discrimination and Stigma scale (DISC-12) [43]; Human Development Index (HDI) [44]; ISMI scale [41]	Two-thirds (62.5%) of participants reported anticipated and/or experienced discrimination in the workplace related to their mental health problems. Almost 60% of participants have stopped themselves from applying for work or education because of anticipated discrimination. Participants in countries with a very high HDI reported significantly more often anticipated or experienced discrimination than participants in countries with moderate/low HDI. Experienced workplace discrimination was less frequently reported than anticipated discrimination and was independently associated with unemployment. Unemployment was also associated with lower education and admission to a psychiatric hospital	6/7 QA items Bias of non-response can’t be assessed (non-response rate not reported)
Corker et al. (2014) [33]	Cross-sectional survey	People diagnosed with major depression (25) or schizophrenia (23) in the previous year in seven countries	DISC-12 scale [43]	Participants with depression reported more discrimination in regard to neighbours, dating, education, marriage, religious activities, physical health and acting as a parent than participants with schizophrenia. Participants with schizophrenia reported more discrimination with regard to the police compared to participants with depression	7/7 QA items
Krajewski et al. (2012) [34]	Cross-sectional	People with one or more psychiatric diagnosis in six countries (104)	PDD scale [40]; ISMI scale [41]; BUES scale [42]: only self-efficacy/self-esteem (SESE) and power/powerlessness (PP) subscales were used	In Romania, self-stigma and PDD were inversely correlated, while SESE was not a significant predictor of self-stigma as compared to other countries Significant predictors of high self-stigma were: being aged 50–59 years; being employed; having poor social support; and minimal-to-low SESE and PP scores	6/7 QA items Bias of non-response can’t be assessed (non-response rate not reported)
Zlati et al. (2011) [22]	Cross-sectional	High-school students (232)	Level of Familiarity Questionnaire [45]; Questions extracted from the National Survey of Mental Health Literacy in Young People in reference to a vignette, which depicted an individual with typical symptoms of schizophrenia [46]; PDD scale [40]; Attribution Questionnaire [47]; Treatment Seeking Behaviour Scale [48]	Adolescents have a low level of mental health literacy and have difficulties recognizing schizophrenia misidentifying it as depression. Teenagers are aware of the existing stereotypes and believe that most people stigmatize individuals with mental illnesses	6/7 QA items Bias of non-response can’t be assessed (non-response rate not reported)
Popescu et al. (2015) [23]	Controlled design with pre and post evaluation Control group; No educational intervention or contact	First and sixth year medical students (280)	Vignettes from the MacArthur Mental Health Social Survey model covering four case vignettes (alcohol addiction, major depressive disorder, schizophrenia and a control- a mentally healthy individual that goes through hardships lately) [38] PDD scale [40] were used to assess the attitudes of medical students towards mental illness	Statistically significant improvements in attitudes towards people with mental illness after an educational intervention and contact with psychiatric patients No significant change was observed in the control group	6/7 QA items Bias of non-response can’t be assessed (non-response rate not reported)
Popescu et al. (2017) [24]	Cross-sectional	First year Romanian and international medical students (322)	PDD scale [40] Vignettes including alcohol addiction, depression and schizophrenia were used to evaluate knowledge about mental illnesses	Medical students reported negative attitudes towards people with mental illness and a moderate desire for social distance. Romanian medical students as compared to their international colleagues, express more desire for social distance and higher perceived violence towards people with alcohol addiction. International students have better knowledge regarding mental illness compared to Romanian students. Familiarity with mental illness was associated with lower levels of desire for social distance	7/7 QA items
Todor et al. (2012) [25]	Cross-sectional	College students in Economics, Social work and Theology (150)	Opinions about Mental Illness Scale – evaluate the opinions and attitudes regarding etiology, treatment and prognosis of mental illness [49]. The scale consist of five subscales: authoritarianism (the opinion that people with mental illnesses cannot be held accountable for their acts and they should be controlled by society) benevolence (an attitude that could be placed between tolerance and pity/compassion) mental hygiene ideology (the opinion that mental illness is similar to other illnesses and it should be treated adequately by specialists) social restrictiveness (the opinion that mentally ill people should be restricted in some social domains) interpersonal etiology (the belief that the real cause of a mental illness is problematic interpersonal relationships)	Students held stigmatizing attitudes towards people with mental illness reporting the highest scores in authoritarianism, benevolence and mental hygiene ideology subscales. Theology students scored highest in authoritarianism All participants registered high scores in Social Restrictiveness reflecting attitudes of intolerance towards individuals with mental illness	6/7 QA items Bias of non-response can’t be assessed (non-response rate not reported)
Vogel et al. (2017) [26]	Cross-sectional	College students from ten countries (226)	Self-Stigma of Seeking Help Scale [50]; Stigma Scale for Receiving Psychological Help [51]; Attitudes Toward Seeking Professional Psychological Help Scale–Short Form [52];	Self-stigma mediated the relationship between public stigma and attitudes toward seeking psychological help among college students in each country. The relationship between public and self-stigma was weaker in Romanian students compared to students in other countries	6/7 QA items Bias of non-response can’t be assessed (non-response rate not reported)
Voinescu et al. (2010) [27]	Cross-sectional	Medical residents (112)	A questionnaire described by Balon et al. assessing the attitudes towards psychiatry, as well as attitudes of health professionals towards psychiatric patients. [53]	Although psychiatry is perceived to be lower in status compared to other residency programmes, the majority of medical residents also think psychiatry is a rapidly expanding, and valid medical branch that has made important progress in addressing mental illness Psychiatry as well as mental illness is still significantly stigmatized in Romania. More than half (52%) of medical residents agreed that they felt uncomfortable with mentally ill patients, while the same proportion stated they have been discouraged by family, colleagues and mentors in choosing psychiatry in their medical training. Moreover, most trainees agreed that those who have not been admitted in other residency programmes would eventually chose psychiatry	6/7 QA items Bias of non-response can’t be assessed (non-response rate not reported)
Mihai et al. (2007) [28]	Cross-sectional	Medical students, medical personnel working in psychiatric settings, healthcare professionals (-)	Attitudes and beliefs about people with schizophrenia	Healthcare professionals held negative attitudes towards people with mental illness and especially towards people with schizophrenia The majority of healthcare professionals avoid contact with mentally ill patients, arguing they lack skills in approaching these patients	6/7 QA items Bias of non-response can’t be assessed (non-response rate not reported)
Tirintica et al. (2018) [35]	Cross-sectional	Mental health service (MHS) users Caregivers (close family members) Mental health providers in three east European countries (200)	A modified version of WHO Pathway Encounter Form questionnaire [54] was used to evaluate the delay to the first psychiatric consultation measured as the time between first appearance of symptoms and first contact with MHS A second questionnaire assessed factors such as: mental health knowledge, attitudes towards psychiatry, mental illness or help-seeking, and social support. The second questionnaire was applied to patients, care givers and mental health providers	Family was the one requesting a psychiatric consultation in 50% of cases in Romania and almost half accessed MHS directly. The majority of patients had a delay of up to 3 months, with 22% of them presenting with more than one year delay while under 10% sought help as soon as symptoms occurred. Longer delays were reported by those who accessed MHS through a doctor’s recommendation and had a diagnosis of alcohol addiction or psychotic disorder Negative attitudes towards mental illness or seeking professional help were associated with longer delays in contrast to having good social support, which was associated with shorter delays	6/7 QA items Bias of non-response can’t be assessed (non-response rate not reported)
Beldie et al. (2012) [36]	Descriptive	Description of anti-stigma interventions by mental health experts in 14 European countries, using all available evidence regardless whether it was published or not General population, high-school students, journalists (-)	-	Four anti-stigma projects took place in different regions of Romania targeting the general population and subgroups such as high-school students and journalists. Campaigns involved people with lived experience of metal illness and used media channels to promote positive attitudes towards people with mental illness and better knowledge regarding mental health. No empirical evidence was published regarding these actions due to finacial reasons and a lack of personnel	7/7 QA items

#### Public beliefs about people with mental illness

Most of the studies assessing public attitudes towards people with mental illness in Romania are limited to specific subgroups of the population and small samples. However, there are two reports based on representative samples: an European and a national survey of community attitudes towards people with mental illness [29, 30].

According to the European survey, the Romanian population held stigmatizing attitudes about people with mental illness at a slightly higher percentage compared to the European average [30]. Unpredictability and dangerousness stereotypes were the most common, being held by 65%, respectively 45% of the population, while a smaller percentage believed people with mental illness never recover (20%) and are to blame for their illness (14%) [30].

The national community research showed that the perceived likelihood of violence varied with the disorder, being highest for alcohol addiction followed by schizophrenia [29]. A similar pattern is observed also for the desire for social distance. Whereas the person presenting depression in the vignette was accepted, as being considered in need of help, people presenting alcohol addiction and schizophrenia in the vignettes were rather rejected being considered dangerous [29]. Using the Perceived Devaluation-Discrimination (PDD) scale, which asks about the general/community perception, the same study reports strong negative attitudes of discrimination towards people with mental illness in all life areas, especially in the labor and marital market. Almost two-thirds of the population share the view that most people do not respect, take seriously or trust a person who has been admitted to a psychiatric hospital [29]. Half consider that most people view admission to the psychiatric hospital as a personal failure; believe these persons are less intelligent and would not accept such persons as their friends [29]. The results from these two representative studies described above are in part replicated in other four studies on specific samples such as teenagers [22] and college students [23–25]. Adolescents as well as students believe most people stigmatize individuals with mental illness. Furthermore, they marginally endorsed stereotypes of dangerousness and responsibility. Attitudes of prejudice and discrimination towards the psychiatric patient were observed also in healthcare professionals [27–29]. The majority of medical doctors interviewed in a study and more than half (52%) in another study, agreed they feel uncomfortable with the psychiatric patient [27, 28]. Similar findings are reported by a qualitative research on a representative sample of 98 mental health professionals who stated that healthcare specialists avoid coming in contact with the psychiatric patient leading to neglect and discrimination [29].

#### Attitudes towards help-seeking

Attitudes toward help-seeking were examined in three studies including a representative sample of the general population aged 18 and over [29], a sample of high-school students [22], and a sample of university students [26]. The general population is inclined towards non-professional help, especially discussing with family and friends, when dealing with a mental health problem. Consulting a psychologist, social worker or priest is preferred over medical treatment and admission into a psychiatric facility. It is not preferred however, over consulting a general practitioner. Consulting a psychiatrist, taking medication and admission into a psychiatric hospital, are considered more appropriate in a significantly greater extent for the vignette describing schizophrenia compared to those describing major depressive disorder and alcohol dependence [29]. High-school students are willing to seek help in the form of family care, psychological treatment and folk medicine. They are not willing to seek professional treatment. Higher rates of willingness to seek help in any form were associated with endorsing the responsibility stereotype (individuals are responsible for their mental illnesses) [22]. Public and self-stigma were associated with more negative attitudes towards help-seeking in a sample of Romanian university students [26]. The most important factors influencing the delay in accessing mental health services were public stigma and lack of knowledge regarding mental health and available treatments [35].

#### Perceived, anticipated and experienced stigma and discrimination, and self-stigma

Four studies report outcomes on patients’ perception of stigma and discrimination [31–34] Discrimination in the workplace was anticipated or experienced by more than half of one study sample with major depression [32] and almost 60% of them had stopped themselves from applying for work, education or training because of anticipated discrimination. A third of mental health service users reported moderate to high levels of self-stigma and two-thirds perceived moderate to high levels of public stigma towards patients with mental illness [34]. Patients with a first episode of major depression reported discrimination to a greater extent and in more life areas than patients with a first episode of psychosis [33]. Patients with depression reported more discrimination regarding education, marriage, religious activities, neighbours and acting as a parent while patients with psychosis reported more discrimination with regard to the police. Lower rates of self-stigma and perceived discrimination were associated with less stigmatizing public attitudes, higher rates of help-seeking and better perceived access to information [31].

#### Structural stigma

No study explicitly assessed manifestations of structural stigma [55]. However, the opinion of mental health specialists about the stigma associated with the healthcare system in Romania clearly identified stigma at this level [29, 36]. They described lack of funds and poor management of existing resources, inefficient coordination between institutions of the mental healthcare system and underdevelopment of community services [29, 36]. According to the majority of mental health specialists consulted in the study, there are a few weak spots in the healthcare system in Romania leading to stigmatization and discrimination of patients with mental illness. Prevention is considered to be a major problem of the system. This aspect is almost completely neglected on the path to care followed by a person with mental health problems [29]. In consequence, patients usually present to the doctor when the disorder is severe and recovery is difficult. The mental health community network is another weak spot mentioned. The community network is severely underdeveloped in Romania with an impact on the quality of care. Due to a lack or insufficiency of community resources, the follow-up of patients is difficult, and not much is done for integrating them back into the community [29, 36].

### Socio-demographic and other factors associated with mental health related stigma in Romania

Stigma has been conceptualised as one aspect of mental health literacy as defined by Kutcher et al. [37]. Low mental health literacy was associated with low educational level and age group 50–59 years [29]. Perceived risk of violence, and higher perceived devaluation but not discrimination are greater in people with high media consumption [29]. Perceived discrimination and devaluation are reported to be higher in low socio-cultural settings and higher discrimination was reported in Dobrogea, Banat, and Oltenia regions and lower in Muntenia, Maramures and Moldova regions [29]. Experienced workplace discrimination was independently positively associated with unemployment [32]. Being employed and having a university educational degree as well as having proper social support were considered to be protective factors because of their association with less self-stigma [32, 34].

### What differences does comparative research on stigma including Romania suggest?

The Romanian population has comparable knowledge about mental illness to the American population [29, 56] and a similar pattern of preferences regarding treatment, with both countries opting rather for non-biological, non-specialized treatment. Perceived public stigma and discrimination against people with mental illness are significantly more severe and cover more life areas in Romania compared to other regions in Germany, Slovakia, and Russia [29, 57]. Results from a study conducted in six European countries showed that self-stigma is less frequent in Romanian population compared to Croatia, Malta, and Lithuania but more frequent than in the Swedish population [34]. Similarly, perceived public stigma was reported to be less frequent in Romania compared to the other European countries in the study [34]. The same study showed an inverse association between perceived public stigma and self-stigma in the Romanian population compared to other countries in the study, which showed either a positive correlation or no correlation. Self-efficacy/self-esteem was not a predictor of self-stigma in Romania compared to Sweden, Croatia, and Lithuania [34]. The association between public stigma and self-stigma in Romania was weaker than the average of other countries such as the USA, Australia, Canada and the United Arab Emirates [26]. Romanian mental health service users diagnosed with a first episode of mental illness reported less perceived and experienced stigma compared to their correspondents in Poland and Sweden [33].

### Interventions targeting mental health stigma conducted in Romania

One national campaign, locally coordinated, was conducted in the major regions of the country immediately after Romania joined the EU but shortly ceased due to a lack of funds and personnel. There are no follow-up results published regarding the effectiveness of this campaign, but a descriptive paper summarized the campaign using experts’ opinions [36].

The campaign took place between 2007 and 2008 in different regions of the country through local mental health centres and included four major projects: ‘Mental health caravan’, ‘Schizophrenia should not be a reason for discrimination’, ‘Trust my mind! Stop the prejudice against mental illness’ and ‘Confide in their mind’.

The ‘Mental health caravan’ project aimed to inform and educate the general population through presentations, distribution of informative educational materials and a local radio campaign targeting stigma associated with mental illness.

‘Schizophrenia should not be a reason for discrimination’ project was carried out in four high schools in the capital city with the direct involvement of the students. The project’s aim was to increase students’ awareness of mental health issues, allowing them to better understand the difficulties people with mental illness have to face.

The project ‘Trust my mind! Stop the prejudice against mental illness’ focused on the qualities of people with mental illness and their right to work. Activities included poster making, organizing opinion polls and conferences and launching a volume of poetry written by a person with mental illness. The campaign was popularized through mass media.

‘Confide in their mind’ was carried out in six major cities of Romania and involved the press as well as the general population. Presentations focused on the need to form a correct image of people with mental health problems.

## Discussion

This work provides a comprehensive report on stigma related to mental illness in an eastern European context and discusses the most relevant evidence for future anti-stigma interventions. We identified a particularly small number of studies (*N* = 15) relevant to the stigma depiction in Romanian population, in line with the findings of Winkler et al.[1]. Moreover, most of them were published more than ten years ago, drawing attention to a pressing need to refresh and increase the current evidence. The lack of behavioural outcomes and that of objective evidence for the delivery and effectiveness of anti-stigma interventions are also critical findings to be addressed. A recent review of stigma in LMICs raised similar points as a priority for future research [58].

In Romania, stigma is present to a significant extent at all levels. Structural stigma as well as public stigma affects people with mental illness on a regular basis leading to negative attitudes towards help-seeking and delayed access to mental health services. A particularly harmful negative stereotype held frequently against people with mental illness in Romania is that they are unpredictable and dangerous [29–31]. Moreover, perceived likelihood of violence was associated with high media consumption [29] suggesting an important role of mass media in shaping the picture of the mentally ill patient. Studies have consistently confirmed that both entertainment and news media depict remarkably dramatic and distorted images of mental illness that emphasize unpredictability, dangerousness, and criminality [59–61]. The impact on individuals with mental illness is detrimental contributing to low self-esteem, experienced discrimination and unwillingness to seek help [62]. On the other hand, there is evidence that media can be used effectively in challenging public stereotypes and discrimination and advocating for a positive image of people with mental health problems [63]. Social media channels and tools are increasingly used in anti-stigma interventions to influence public attitudes towards people with mental illness [64]. Offering a space to share a personal unheard story with many people, beyond the limitations of face-to-face interactions, can create a sense of empowerment and solidarity. Social media channels can also enhance communication and learning between professionals as well as service users [64]. Results for effective anti-stigma interventions focused on mass media include contact-based educational methods targeted to journalists and implementation of mental health educational modules in the training of media workers [65].

Interestingly, perceived public stigma was present in a higher proportion than self-stigma in persons with mental illness [31–34]. However, their relationship and the impact on help-seeking behaviour generated mixed results. Help-seeking attitudes and behaviours in the Romanian population seem to be influenced mainly by public stigma [33] while its relationship with self-stigma was weaker compared to other cultural settings [26, 34]. The internalization of public stigma into self-stigma seems to be less pronounced in Romanians suggesting that other factors may interact in the development of self-stigma. Compared to other European countries, self-efficacy and self-esteem were not significant predictors of self-stigma in the Romanian population [34]. The hypothesis that low self-efficacy and self-esteem are components or consequences of self-stigma [66, 67] is not supported by the evidence presented in the current review. Overall, the results suggest that self-stigma may be more context-dependent than person-dependent. The sociocultural setting could affect differently how individuals apply the publicly held messages to themselves. Further research is needed to investigate in more detail what and how socio-cultural factors influence mental health stigma in Romania and carefully addressed them in mental health policy formulation and anti-stigma interventional programs. Another important finding of current work is that proper social support may play a protective role against self-stigma [34]. Along with other evidence [68] to support our findings, these results highlight the important role played by peer support in enhancing empowerment and decreasing self-stigma and underline its potential usefulness in interventional programmes.

While self-stigma was less present in the Romanian psychiatric population than perceived stigma and discrimination [34], its impact in the work setting has proved to be important [32]. Other major studies [66, 69] had similar results, implying a worldwide concern and a clear need to consider work-related stigma and discrimination a priority for future research and intervention. Besides targeting public stigma particularly in employers, the results of our review suggest an intervention should also focus on the self-stigma of the employees.

Further, anticipated discrimination was not only more frequently reported than experienced discrimination in the workplace but other studies have proved that they many not necessarily be associated [70, 71]. Our results indicated other factors may be involved as anticipated discrimination was associated with self-stigma and led to avoiding behaviour such as reluctance to apply for work or education [32]. Corrigan explains the dynamics between self-stigma, anticipated discrimination and avoidance behaviour with a phenomenon called the “why try” effect [72]. The phenomenon describes a tendency that appeared in those with self-stigma and anticipated discrimination to avoid high-risk situations. On the other hand, studies on employers have indicated that they display indeed a marked tendency to have negative attitudes towards people with mental health problems [73–75]. Although improvements in employers’ awareness, knowledge, and attitudes are reported in places where coordinated anti-stigma actions were conducted, especially in high-income countries [76–78], workplace stigma remains a present concern, significantly affecting people with mental illness, particularly in LMICs.

Our findings along with others have indicated that an alarming level of stigma is also present in healthcare or mental healthcare professionals [79–82]. Despite of evident superior knowledge of mental illness in mental health professionals compared to other health specialist, they are nevertheless predisposed to negative cultural stereotypes. Studies have shown that up to 50% of mental health service users reported discriminatory behaviours in mental health specialists [80–82]. The overwhelming evidence highlights the imperative need to consider mental health professionals as a priority target for anti-stigma interventions. A few anti-stigma actions have been conducted in the last fifteen years in Romania with international support and being locally coordinated in the absence of a common conceptual framework. The only available data regarding these interventions consist of a mental health expert’s description of these projects [36]. The findings underline two main problems: the lack of a conceptual framework for working with community stigma and a very low rate of publication. Both problems can be seen as consequences of structural stigma and may be attributed, at least in part, to poor allocation of funds to mental health compared to other medical specialities. Implications are numerous, leading to uncoordinated, uncommunicated and one-time actions with low potential long-term effectiveness. All these translate into more stigma and discrimination towards people with mental illness.

### Strengths and limitations

An important limitation of the current study is the exclusive focus on the local context of Romania thus the recommendations may be less generalizable elsewhere. Another is the small amount of evidence to review with an overall qood quality of reporting. Important aspects to be improved in order to increase quality in future research concerns especially setting,sample and response rate reports. A further limitation is the relative small number of databases searched. We cannot rule out the possibility that some potentially relevant papers might have been missed. However, as there was a considerable overlap between the search results of the three databases, it is unlikely that this is a significant problem. Moreover, our search results are in line with those reported by other seminal research, indicating a scarcity of empirical evidence regarding stigma in Romania [1]. We are also aware of the limitation that most of the included studies are more than ten years old, potentially limiting the understanding of current state of affairs. Taken together, these facts emphasize the pressing need for more and recent empirical evidence regarding stigma related to mental illness in Romania in order to design and deliver targeted and effective anti-stigma actions. Despite the limitations, this review meets a major need emphasized in recent research by contributing to the gap of evidence concerning Eastern European countries. To the best of our knowledge, this is the first review of literature regarding stigma related to mental illness in Romania and the first attempt to make evidence-based recommendations for future research and intervention.

### Implications for research, policy and practice

In meeting the needs emphasized by the current review and in light of the recommendations of global stigma research [63, 66, 82, 83], interventions in Romania should target three main dimensions of stigma and discrimination: public stigma as in knowledge, attitudes and behaviours; self-stigma as the internalization of public beliefs followed by anticipated discrimination; and structural stigma in the form of legal and policy frameworks.

#### Public and self-stigma

As an evidence-based recommendation for the approach of public stigma, the intervention should consider several aspects. It should target priority groups of the Romanian population: healthcare and mental health personnel, family members of people with mental illness, employers and journalists. The intervention should aim to change the community’s attitudes towards people with mental illness through activities centred on forming an accurate image and an understanding of the extent of the stigma they carry. Regions with lower socio-economic status should be a priority focus as mental illness is frequent in these groups while knowledge is poor and perceived stigma and discrimination is higher compared to other groups [29, 84]. They may benefit especially from educational activities focused on enhancing mental health awareness and communication. Short and medium-term interventions both showed an improvement in public attitudes towards people with mental illness [85, 86] although the scale and duration of such interventions are still debated. Involving people with mental health problems who experienced stigma and discrimination in the intervention, as ‘experts by experience’, represents a general evidence-based recommendation being considered the most effective way in changing public attitudes and enhancing the effects of community educational measures [87]. Using mass-media both as a target for stigma reduction and as an efficient means for improving attitudes towards mental illness in the general population has already proved to be beneficial in other socio-cultural settings [64, 65, 88] and was also emphasized in this work. Anti-stigma interventions can benefit from targeting media workers as potential social influencers in stigma advocacy and using social media channels in promoting a correct image of people with mental illness [89]. More promising lines of action in fighting community stigma include collaboration with formal and informal educational institutions and NGOs and targeting other potential anti-stigma agents such as priests and other community leaders with greater cultural impact on the local communities.

Interventions should also consider help-seeking behaviours, being a major issue in the Romanian population. Public stigma proved to play a major role independent of self-stigma, suggesting interventions should consider a specific approach targeting people’s attitudes towards those admitted to a psychiatric hospital in particular. Further research should answer how mental health service users in Romania perceive the mental health system in terms of accessibility, efficiency, and stigma as it may also influence their help-seeking behaviour.

Stigma related to the work environment was another hot topic for the Romanian population. Two main issues have emerged from this analysis: employment of people with mental illness and their rights in the workplace. Thus, interventions should aim to improve both sides of employers’ attitudes: towards hiring people with mental health problems and attitudes towards those already hired. As a particular aspect, self-stigma seemed to have a significant impact in the workplace, contributing to anticipated discrimination and avoidance behaviour. Although low self-esteem and self-efficacy may not predict self-stigma in the general psychiatric population, when it comes to the workplace, interventions may benefit from empowering people with mental illness and increasing their self-esteem and self-efficiency in the workplace [66, 67, 69, 70].

Self-stigma associated with mental illness has not been sufficiently assessed in Romanian population. There are very few and mixed data picturing an incomplete view. Further research is needed to explore how and to what extent self-stigma may interact with public beliefs in hindering people’s access to mental care services. Self-stigma can be handled by training mental healthcare professionals to educate and empower the person with mental health problems [67]. The problem addresses structural changes in policies regarding medical education and is discussed in more detail below.

Mental health professionals have been identified not only as a priority target for anti-stigma reduction but as anti-stigma agents as well [90, 91]. Studies have agreed that health specialists can represent efficient means of reducing discrimination and advancing anti-stigma efforts through advocating for structural and legal changes [92–95]. Anti-stigma advocacy training is not currently part of the general training of mental health professionals or medical students. Growing evidence emphasizes their potential effectiveness in promoting mental health and reducing self-stigma in patients as well as addressing public stigma in families, other health specialists or the general population. It is not yet clear how anti-stigma advocacy may be best included at the structural level in the education of mental health specialists and more evidence coming from mental health settings is needed in Romania. A recent systematic review [95] assessing the effectiveness of training in health advocacy and anti-stigma competency concluded that intervention in mental healthcare professionals should check a few important points: integrate advocacy in their professional roles; be developed based on a situational analysis; include people with experienced stigma and discrimination; and use behavioural outcomes. Research and educational fields should work together in the building and delivery of evidence-based actions to secure both educational and legal frameworks.

#### Structural stigma

This work has drawn attention to several pressing needs of the mental health system in line with those addressed above. As concluded in this work, there are no integrated conceptual frameworks of action or specific practical guidelines in most fields of anti-stigma intervention. For instance, there are no evidence-based tailored intervention guidelines in working with community stigma and discrimination, or as stated above, no particular interest in integrating anti-stigma advocacy training in medical education and psychiatric training curricula, although there are many benefits already proved [93, 94, 96, 97]. Some efforts have been made by Romanian mental health experts [21] in proposing a biopsychosocial human rights conceptual framework that could meet some of the problems faced in the community field. The most effective Romanian anti-stigma actions came from the collaboration of mental health professionals with human rights NGOs as the Center of Legal Resources, covering multiple approaches [29]. Another pressing need of the mental health system is to create an efficient community network of specialists ready to address the integration and anti-stigmatization of people with mental illness. Actions to support this endeavour target policy change and training mental health specialists to fight for stigma reduction.

## Conclusions

This review was conducted based on current stigma research recommendations to help fill the evidence gap concerning post-soviet European countries. The level of both public and structural stigma is high in Romania, leading to negative attitudes towards help-seeking and delayed access to mental health services. Stigma is present at all levels affecting in particular work and marital life areas and is highest towards those with a diagnosis of schizophrenia or who have been admitted to a psychiatric institution. A few anti-stigma initiatives targeting the general population were conducted in Romania but without proper evaluation, follow-up or scientific dissemination. Based on the results highlighted in this review, a few lines of action should be prioritize in Romania. Increase high-quality research in order to develop evidence-based tailored interventions targeting priority subgroups of the population. Ensure proper evaluation and scientific dissemination. Elaborate and adopt guidelines for better addressing public stigma and advocate for policy changes. Training mental health personnel to take up more of these roles can be a good first step in fighting stigma associated with mental illness in Romania.

### Supplementary Information

**Additional file 1.** PRISMA checklist.

**Additional file 2.** Search strategy.

**Additional file 3.** MMAT QA.

## Data Availability

The datasets used and/or analysed during the current study available from the corresponding author on reasonable request.

## Abbreviations

NGO: Non-governmental organization
EU: European union
MMAT: Mixed methods assessment tool
