# Supplementary material for: Mental health related stigma in Romania: systematic review and narrative synthesis
Source: BMC Psychiatry. 2023 Sep 8;23:662. doi: 10.1186/s12888-023-05147-3 (PMC10486137; doi:10.1186/s12888-023-05147-3)
Supplement: Supplementary file 2 — Additional file 2. Search strategy. [file 12888_2023_5147_MOESM2_ESM.pdf]

ADDITIONAL FILE 2: SEARCH STRATEGY

PubMed

Concept	Description of concept	Research Terms
A	Stigma	Stigma* [Mesh] OR stigma* [TW] OR stigma* [tiab] OR knowledge [Mesh] OR knowledge [TW] OR knowledge [tiab] OR awareness [Mesh] OR awareness [TW] OR awareness [tiab] OR discriminat* [Mesh] OR discriminat* [TW] OR discriminat* [tiab] OR prejudic* [Mesh] OR prejudic* [TW] OR prejudic* [tiab] OR social exclusion [Mesh] OR social exclusion [TW] OR social exclusion [tiab] OR social perception [Mesh] OR social distance [Mesh] OR social distance [TW] OR social distance [tiab] OR social perception [TW] OR social perception [tiab] OR public opinion* [Mesh] OR public opinion* [TW] OR public opinion* [tiab] OR stereotyp* [Mesh] OR stereotyp* [TW] OR stereotyp* [tiab] OR myth [Mesh] OR myth [TW] OR myth [tiab] OR attitude* [Mesh] OR attitude* [TW] OR attitude* [tiab] OR label* [Mesh] OR label* [TW] OR label* [tiab] OR misconception* [Mesh] OR misconception* [TW] OR misconception* [tiab] OR literacy [Mesh] OR literacy [TW] OR literacy [tiab] OR behaviour* [Mesh] OR behaviour* [TW] OR behaviour* [tiab] OR intended behaviour* [Mesh] OR intended behaviour* [TW] OR intended behaviour* [tiab] OR avoid* [Mesh] OR avoid* [TW] OR avoid* [tiab] OR reject* [Mesh] OR reject* [TW] OR reject* [tiab] OR *toleran* [Mesh] OR *toleran* [TW] OR *toleran* [tiab] OR anti-stigma intervention* [Mesh] OR anti-stigma intervention* [TW] OR anti-stigma intervention* [tiab] OR stigma reduction intervention* [Mesh] OR stigma reduction intervention* [TW] OR stigma reduction intervention* [tiab] OR intervention* [Mesh] OR intervention* [TW] OR intervention* [tiab] OR fight* [Mesh] OR fight* [TW] OR fight* [tiab]
B	Mental disorders	Mental disorder* [Mesh] OR mental disorder* [TW] OR mental disorder* [tiab] OR mental health [Mesh] OR mental health [TW] OR mental health [tiab] OR mental healthcare [TW] OR mental health service* [TW] OR mental disease* [Mesh] OR mental disease* [TW] OR mental disease* [tiab] OR mental* ill* [Mesh] OR mental* ill* [TW] OR mental* ill* [tiab] OR psychiatric disorder* [Mesh] OR psychiatric disorder* [TW] OR psychiatric disorder* [tiab] OR psychiatric disease* [Mesh] OR psychiatric disease* [TW] OR psychiatric disease* [tiab] OR psychiatric condition* [Mesh] OR psychiatric condition* [TW] OR psychiatric condition* [tiab] OR psychological* condition* [Mesh] OR psychological* condition* [TW] OR psychological* condition* [tiab] OR psychological* problem* [Mesh] OR psychological* problem* [TW] OR psychological* distress* [TW] OR emotional problem* [TW] OR emotional distress* [TW] OR depressi* [Mesh] OR depressi* [TW] OR depressi* [tiab] OR schizophrenia

		[Mesh] OR schizophreni* [TW] OR schizophreni* [tiab] OR psycho* [Mesh] OR psycho* [TW] OR psycho* [tiab] OR anxiet* [Mesh] OR anxiet* [TW] OR anxiet* [tiab] OR obsessi* [TW] OR obsessi* [Mesh] OR obsessi* [tiab] OR bipolar* [tiab] OR bipolar* [Mesh] OR bipolar* [TW] OR mani* [TW] OR mani* [Mesh] OR mani* [tiab]
C	Romania	Romania* [tiab] OR Romania* [TW]

Search strategy « A » & « B » & « C »

Web of Science

Concept	Research Terms
Stigma related to mental illness	**TOPIC:** (stigma* OR knowledge OR awareness OR discriminat* OR orejudic* OR social exclusion or social perception OR social distance OR public opinion OR stereotyp* OR myth OR attitude* OR label* OR misconception* OR literacy OR behaviour* OR intended behaviour OR avoid* OR reject* OR *toleran* OR anti-stigma intervention* OR stigma reduction intervention* or intervention* OR fight*) AND (mental disorder* OR mental disease* OR mental* ill* OR psychiatric disorder* OR psychiatric disease* OR psychiatric condition* OR psychological condition* OR psychological problem* psychological distress* OR emotional problem* OR emotional distress OR depressi* OR schizophreni* OR psycho* OR anxiet* OR obsessi* OR bipoolar* OR mani* OR mental health OR mental healthcare OR mental health service*) AND Romania*
