# Supplementary material for: Mental health related stigma in Romania: systematic review and narrative synthesis
Source: BMC Psychiatry. 2023 Sep 8;23:662. doi: 10.1186/s12888-023-05147-3 (PMC10486137; doi:10.1186/s12888-023-05147-3)
Supplement: Supplementary file 3 — Additional file 3. MMAT QA. [file 12888_2023_5147_MOESM3_ESM.pdf]

Additional file 3 – Quality assessment using Mixed Methods Assessment Tool (MMAT)

MMAT QUALITY ASSESSMENT ()	Stanculescu et al [29]	Eurobarometer survey[30]	Evans-Lacko et al [31]	Brouwers et al [32]	Corker et al [33]	Krajewski et al.[34]	Zlati et al.[22]	Popescu et al.[23]	Popescu et al.[24]	Todor et al.[25]	Vogel et al.[26]	Voinescu et al.[27]	Mihai et al.[28]	Tirintica et al.[30]	Beldie et al.[36]
**Screening questions (for all types)**															
Are there clear research questions?	✓	✓	✓	✓	✓	✓	✓	✓	✓	✓	✓	✓	✓	✓	✓
Do the collected data allow to address the research questions?	✓	✓	✓	✓	✓	✓	✓	✓	✓	✓	✓	✓	✓	✓	✓
**Qualitative studies**															
Is the qualitative approach appropriate to answer the research questions?	-	-	-	-	-	-	-	-	-	-	-	-	-	-	✓
Are the qualitative data collection methods adequate to address the research question?		-	-	-	-	-	-	-	-	-	-	-	-	-	✓
Are the findings adequately derived from the data?	-	-	-	-	-	-	-	-	-	-	-	-	-	-	✓
Is the interpretation of results sufficiently substantiated by data?	-	-	-	-	-	-	-	-	-	-	-	-	-	-	✓
Is there coherence between qualitative data sources,	-	-	-	-	-	-	-	-	-	-	-	-	-	-	✓

collection, analysis and interpretation?															
**Quantitative descriptive studies (descriptive cross-sectional, survey)**															
Is the sampling strategy relevant to address the research question?	-	✓	✓	✓	✓	✓	✓	✓	✓	✓	✓	✓	✓	✓	-
Is the sample representative of the target population?	-	✓	✓	✓	✓	✓	✓	✓	✓	✓	✓	✓	✓	✓	-
Are the measurements appropriate?	-	✓	✓	✓	✓	✓	✓	✓	✓	✓	✓	✓	✓	✓	-
Is the risk of nonresponse bias low?		✓	✓	Can’t tell	✓	Can’t tell	Can’t tell	Can’t tell	✓	Can’t tell	Can’t tell	Can’t tell	Can’t tell	Can’t tell	-
Is the statistical analysis appropriate to answer the research question?	-	✓	✓	✓	✓	✓	✓	✓	✓	✓	✓	✓	✓	✓	-
**Mixed methods** Is there an adequate rationale for using a mixed methods design to address the research question?	✓	-	-	-	-	-	-	-	-	-	-	-	-	-	-
Are there different components of the study effectively integrated to answer the research question?	✓	-	-	-	-	-	-	-	-	-	-	-	-	-	-
Are the outputs of the integration of qualitative and quantitative components	✓	-	-	-	-	-	-	-	-	-	-	-	-	-	-

adequately interpreted?															
Are divergences and inconsistencies between quantitative and qualitative results adequately addressed?	✓	-	-	-	-	-	-	-	-	-	-	-	-	-	-
Do the different components of the study adhere to the quality criteria of each tradition of the methods involved?	✓	-	-	-	-	-	-	-	-	-	-	-	-	-	-

MMAT:Mixed Methods Assessment Tool
